# RETRACTED: Ye et al. Drug Resistance and Molecular Characteristics of Carbapenem-Resistant OXA-48-Producing *Klebsiella pneumoniae* Strains in Hainan, China. *Microorganisms* 2024, *12*, 49

**DOI:** 10.3390/microorganisms12040661

**Published:** 2024-03-26

**Authors:** Min Ye, Lei Liu, Bin Liu, Xiangdong Zhou, Qi Li

**Affiliations:** 1 International School of Nursing, Hainan Medical University, Haikou 571199, China; minyeai@163.com; 2 Department of Respiratory Medicine, The First Affiliated Hospital of Hainan Medical University, Haikou 570100, China; liulei110052@163.com (L.L.); moxiaoting2022@163.com (B.L.); 3 Hainan Province Clinical Medical Center of Respiratory Disease, Longhua, Haikou 579199, China; 4 NHC Key Laboratory of Tropical Disease Control, Hainan Medical University, Haikou 571199, China

The author retracts the article, ‘Drug Resistance and Molecular Characteristics of Car-bapenem-Resistant OXA-48-Producing *Klebsiella pneumoniae* Strains in Hainan, China’ [1].

Following publication, concerns were raised to the *Microorganisms* Editorial Office that the published paper [1] has significant overlap with a previous publication. The overlap included a number of identical figures and sequence analyses; the previous manuscript had a different authorship to [1].

Adhering to our complaints procedure, an investigation was conducted and the authors admit that there are errors in their article. While the authors cooperated with the Editorial Office during the investigation, they were unable to satisfactorily explain the figure overlap. Consequently, the Editor-in-Chief was unable to confirm the reliability of the findings and subsequently decided to retract this paper.

This retraction was approved by the Editor-in-Chief of the *Microorganisms* journal.

The authors agreed to this retraction.
